# Rural women's preferences for cervical cancer screening via HPV self-sampling: a discrete choice experiment study in chidamoyo, Hurungwe District, Zimbabwe

**DOI:** 10.1016/j.xagr.2024.100414

**Published:** 2024-10-17

**Authors:** Mathias Dzobo, Tafadzwa Dzinamarira, Michael Strauss, Tivani Mashamba-Thompson

**Affiliations:** 1 School of Health Systems and Public Health, Faculty of Health Sciences, University of Pretoria, Pretoria, South Africa (Dzobo, Dzinamarira, and Mashamba-Thompson); 2 Health Economics and HIV/AIDS Research Division (HEARD), University of KwaZulu Natal, Durban, South Africa (Strauss)

**Keywords:** cervical cancer screening, HPV self-sampling, preference, rural women, Zimbabwe

## Abstract

**Background:**

Cervical cancer screening using HPV self-sampling presents a valuable opportunity to enhance access for underserved and never-screened women in Zimbabwe. However, to ensure the successful implementation of this innovative approach, it is crucial to understand the preferences of key stakeholders, particularly women, with regard to the various components of an HPV self-sampling intervention.

**Objective:**

This study aimed to elicit rural women's preferences for HPV self-sampling.

**Study design:**

A DCE questionnaire was administered to 215 women in Chidamoyo, Hurungwe Rural District. Women were asked to choose between two hypothetical screening choices defined by education, location of services, supervision of self-sampling, comfort of sampling device, results notification and care after HPV results. Data were analysed using fixed and mixed logistic regression models.

**Results:**

Results indicated that the comfort of the sampling device had the most significant impact on women's preferences for HPV self-sampling. Women prioritised facility-based self-sampling, female-supervised self-sampling, and face-to-face education on cervical cancer and screening methods. The methods of results notification and care after HPV results did not significantly impact women's choices. The mixed effects results showed preference heterogeneity in some of the attributes. Interaction analyses suggested that preferences were largely homogenous across the following subgroups: never-screened, previously screened, young and older women. The stratified analysis also showed that preferences were consistent among the four subgroups.

**Conclusion:**

Our findings highlight the importance of face-to-face education, comfortable and user-friendly sampling devices, female health worker supervision and health facility-based self-sampling for cervical cancer screening via HPV self-sampling. These insights could guide the design of patient-centric interventions to ensure high uptake and increased screening coverage.

**AJOG Global Reports at a Glance**

**Why was this study conducted?**

• HPV-based cervical cancer screening via self-sampling can potentially increase women's access to screening, resulting in increased cervical cancer screening coverage in Zimbabwe. However, the modalities for offering HPV self-sampling are still to be defined.

• This study explored the HPV self-sampling preferences of women in Chidamoyo, Hurungwe Rural District in Zimbabwe

**Key findings**

• The choice of sampling device had the most significant influence on women's preferences for HPV self-sampling, with women preferring an easy-to-use and comfortable device

• The delivery method for educational information on cervical cancer and screening methods, the gender of the health worker supervising self-sampling, the choice of sampling device and venue for performing self-sampling were important for determining women's preferences

**What does this add to what is known?**

• Our study is one of the few studies and the first in Zimbabwe to explore women's preferences for HPV self-sampling using the discrete choice experiment methodology.

• Implementing education and awareness programmes on cervical cancer and self-sampling is crucial to increase women's acceptability and confidence in HPV-based cervical cancer screening via self-sampling

## Introduction

Cervical cancer remains a significant public health challenge globally. An estimated 660,000 cases and close to 350, 000 deaths of cervical cancer were recorded in 2022. This statistic marks an increase from 604, 000 cases and 342, 000 deaths recorded in 2020, respectively.^1^ Disparities in access to preventive measures like HPV vaccination and cervical cancer screening services exacerbate the disease burden, particularly in low- and middle-income countries (LMICs).^1^

Zimbabwe demonstrates a particularly high cervical cancer burden compared to global averages. Age-standardised incidence and mortality rates for 2022 were estimated at 68.2% and 47.9%, respectively, five times and seven times the global average.^2^ Comparing these statistics with those of a developed country can highlight the gravity of the situation. For instance, Canada had a markedly lower incidence rate of 6.6% and a mortality rate of 2.3% for the same period.^2^ Cervical cancer is the most common malignancy affecting women of childbearing age in Zimbabwe. Without preventive measures such as HPV vaccination and cervical cancer screening, an estimated 5 million women are at risk of developing the disease.^3^ According to the World Health Organisation (WHO), 3528 cases and 2318 deaths due to cervical cancer were reported by the country's Bulawayo and Harare city cancer registries.^2^ The screening coverage in Zimbabwe is very low, with less than 20% of eligible women having been screened.^3^ The low screening coverage is despite the availability of screening services at most of the country's public health facilities.

Visual inspection with acetic acid and cervicography (VIAC) is Zimbabwe's most common screening modality. Despite the government's efforts to provide VIAC screening services in the district and provincial health facilities, screening coverage remains low due to the low uptake of available services.^4^ Several individual, socio-cultural, and health system factors cause the low uptake and utilisation of screening services.^5^ Among them are the lack of knowledge and awareness, inaccessible health services, pain and discomfort of a pelvic examination and societal stigma and discrimination.^5^^,^^6^ HPV testing is a recommended screening method which is highly reproducible and less prone to examiner error. It enhances the cost-effectiveness and efficacy of screening while reducing the burden on healthcare systems by allowing longer screening intervals for HPV-negative women.^7^^,^^8^ Additionally, women can collect cervicovaginal specimens for HPV testing (HPV self-sampling).

HPV self-sampling is an innovative screening method that overcomes some of the barriers associated with a pelvic exam, leading to increased screening coverage. Given that cervical cancer most commonly occurs among never-screened and under-screened women, offering HPV self-sampling presents an excellent opportunity for increasing the participation of women in screening programmes.^9^ The Ministry of Health and Child Care in Zimbabwe is taking a commendable step by implementing HPV testing for primary cervical cancer screening. However, to optimise the implementation of this new approach, understanding the preferences of key stakeholders, particularly women, regarding the various aspects of an HPV self-sampling intervention is crucial. This knowledge will inform policy decisions and guide the design of a successful nationwide screening program.

In this study, we will present different HPV self-sampling delivery approaches to a population of rural women to allow them to determine preferences for different characteristics of HPV self-sampling. A standard method used in preference studies is the discrete choice experiment (DCE). The DCE has been widely used to guide policy design within the healthcare sector, including interventions such as cervical cancer screening.^10^, ^11^, ^12^ To our knowledge, a DCE survey has not previously been used to inform decisions about HPV self-sampling in Zimbabwe. The DCE is beneficial for obtaining quantitative data on preferences for services not yet widely available or implemented in a specific context, e.g. HPV self-sampling-based cervical cancer screening in Zimbabwe. The present study aimed to use a DCE survey to determine preferences for different characteristics of an HPV self-sampling intervention for cervical cancer screening among rural Zimbabwean women. We anticipate that the findings of this research will inform healthcare policy and practice, ensuring that future screening programmes align closely with client preferences.

## Methods

### DCE overview

DCE is a robust survey-based methodology that elicits consumer preferences for goods and services.^13^ The DCE has been widely used in economic research but has recently gained traction within healthcare systems for exploring preferences for healthcare interventions such as HIV self-testing^14^ and differentiated HIV treatment models.^15^ It is underpinned by solid theoretical grounds such as Lancaster's economic theory of value.^16^ It posits that individuals derive utility (or well-being) not from the good itself but rather from the attributes/characteristics of that good.^16^^,^^17^ The attributes of a good or service can take various forms known as levels, and the respondents derived utility changes with each different level of the attribute.^18^

### Study setting

A DCE survey was conducted among a convenient sample of women routinely visiting Chidamoyo Mission Hospital in Hurungwe Rural District. Chidamoyo Mission Hospital is one of the only two district hospitals in the Hurungwe Rural District. The estimated population served by Chidamoyo Mission Hospital is 32,000 people, with approximately 3200 eligible women.^19^ In this study, eligible participants were women aged 18 and older. We chose 18 years because it is the minimal legal age of consent in Zimbabwe. We excluded women who failed to provide written consent to participate in the study.

### Study design

Before conducting the DCE survey, a comprehensive literature review^20^ was conducted for studies published between January 2011 and March 2023. Additionally, formative qualitative research^21^ was carried out in April 2023 using a nominal group technique (NGT). The NGT involved participants ranking the attributes according to their importance or relevance. We identified an initial list of eight characteristics from the literature review and NGT. One attribute was unanimously removed through an iterative process involving an expert panel of public health researchers, gynaecologists and epidemiologists. Using a think-aloud approach, we conducted a pilot study to test the chosen attributes and levels among fourteen purposively sampled women from the target population. After considering comments from the experts, we removed one attribute, and the wording for some of the attributes was changed to make it simpler for the respondents to understand. Supplementary Table 1 shows the final list of attributes agreed upon by the researchers for the DCE study and the definitions used in this study.

### DCE instrument design

Given this study's number of attributes and levels, the resulting choice pairs would be too many and pose a significant cognitive burden to the respondent. Thus, to reduce the number of choice sets, we developed a fractional factorial, unlabeled design of binary choice sets using the D-optimal design in StataBE 18 (StataCorp, College Station, TX).^22^ A D-optimal design ensures that the choice sets (combinations of different levels for each attribute) selected to provide a balance of attributes and levels across the experiment (orthogonality) and that attributes within a choice set never take the same level value (thereby forcing respondents to trade on all attributes and eliciting maximum information).^23^ We generated 32 choice sets, divided into four versions, each with eight choice sets, by including a blocking variable in the design. On recruitment, participants were randomly assigned to one of the four versions.

### Sample size

No standardised method for determining the minimum sample size in DCEs exists. Johnson and Orme recommended a rule-of-thumb: *nta/c ≥ 500*, where *n* = number of respondents, *t* = number of tasks, *a* = number of alternatives per task, and *c* = the largest number of levels for any attribute.^24^ In this DCE, the highest number of levels in any attribute is three, and eight binary choices were presented to each participant (from the total design of 32). A sample size of 94 was calculated using the method by Johnson and Orme. However, to account for the potential loss of participants, we recruited a minimum sample size of 110 participants per subgroup (previously screened/never screened) for a total sample size of 220.

### Eligibility criteria

Participants were eligible for inclusion in the DCE if they (1) were 18 years and older, (2) were residents of Chidamoyo in Hurungwe Rural District, and (3) could read and write Shona. We enrolled participants as young as 18 with no prior screening experience because we were primarily interested in understanding the characteristics of HPV self-sampling, which would make the intervention most acceptable to women.

### Procedures and data collection

Two community health workers (CHWs) sensitised the women in the community regarding the study a few weeks before data collection started during routine public health outreach community programmes. The CHWs explained the study's background and purpose and encouraged women to participate. Data collection took place between March and April 2024. The CHWs were responsible for enrolling study participants who had received treatment or care at the hospital. They did so by providing a detailed explanation of the study's background and purpose. The women signed informed consent forms if they agreed to participate in the study. The CHWs administered paper-based questionnaires in the Shona language to the participants. After completing a short series of socio-demographic and knowledge-related questions, eight choice sets were presented to each participant to respond to by choosing their preferred HPV self-sampling delivery option. Every woman was given a laundry pack containing soap upon completing the questionnaire to incentivise participation in the study. The CHWs illustrated the meaning of choice sets using pictures to improve participants' understanding of the attributes and levels (Supplementary Figure 1).

### Statistical analysis

Individual characteristics were explored descriptively. We estimated participant preferences by initially running a simple fixed-effects logit model (Model 1) and then running a random-effects logit model (Model 2) for the main effects using dummy coding of attribute levels. These models estimate the probability of choosing one alternative over another and are commonly used for estimating model parameters in DCEs that employ a binary design.^15^ Model 1 and Model 2 produced similar results, with the magnitude, direction, and significance of effects broadly consistent. A Hausmann specification test was run to check for violations of the assumption of independence of irrelevant alternatives (IIA) underlying the fixed-effects logit model.^25^ The Hausmann test returned a negative result, indicating that a fixed-effects logit model was more appropriate than a random-effects for estimating preference data. To explore potential preference heterogeneity, we ran the mixed-effects logit model (Model 3) using Halton draws with 1000 replications to estimate the relative utility of each attribute and level. Mixed effects allow for the relaxing of IIA and an assessment of heterogeneity across attributes.^26^ All analyses were conducted in StataBE 18 (StataCorp, College Station, TX).^22^ The results are presented as odds ratios (ORs) in relation to a baseline scenario, which includes the reference levels for each attribute Supplementary Table 1.

### Interaction effects

To investigate heterogeneity based on the variability observed with the mean odds ratio and the standard deviation, we ran fixed effects models to test the interactions between socio-demographic characteristics and the magnitude of preferences for the attributes and levels of HPV self-sampling delivery approaches. For the first interaction model (Model 4) we used a dummy variable (previously screened=1, never screened=0) to investigate heterogeneity between previously screened and never screened women. Model 5 was the interaction model for age, which used the dummy variable (age <30=0, age ≥ 30=1)

To strengthen the methodological foundation of our study, we incorporated the Socio-ecological model as a conceptual framework to underpin the DCE study.^27^ The Socio-ecological model posits that patients and their health-related decisions are influenced by multiple factors: intrapersonal, interpersonal, community, healthcare, and health systems. Each attribute of our DCE, such as the location for performing self-sampling services, the delivery method for educational information on cervical cancer and screening method, supervision of self-sampling, the comfort of the sampling device, notification of HPV results, care and treatment after a positive HPV result align with Socio-ecological model domains. For instance, the preference for female nurse-supervised sampling is tied to the intrapersonal construct of the Socio-ecological model, which may highlight the need for more individual confidence to perform self-sampling without supervision. Using the Socio-ecological model, we can offer a more nuanced interpretation of our findings, highlighting how individual, interpersonal, healthcare, community and health systems factors can shape preferences for HPV self-sampling.

Ethical approval was granted by the Human Research Ethics Committee of the University of Pretoria (548/2022) and the Medical Research Council of Zimbabwe (MRCZ/A/2993). Additional permission was sought and granted by the administration of Chidamoyo Mission Hospital and the Ministry of Health and Child Care in Zimbabwe. All eligible participants provided written informed consent.

## DCE results

### Participant characteristics

A total of 222 women participated in the DCE survey. However, seven women (3.2%) did not complete the correct DCE questionnaire and choice sets and therefore were excluded. Table presents the characteristics of the 215 women included in the study. Our sample (N=215) comprised rural women between 18 and 64 years old. The mean age of our sample was 37.3. Most participants were 30 years and older (139/215; 64.6%), had completed their ordinary levels (114/215;53%), were married (158/215;73.5%), did not go to work (117/215;54.4%) had a net monthly income of less than 50USD (148/215; 69.2%), stayed within 0–5 km from their nearest health facility (113/215; 52.6%), had heard of HPV (185/215; 86.0%) and had been screened for cervical cancer (112/215; 52.1%). Of those screened for cervical cancer (94/112 (83,9%) had been screened within the last five years, and (97/112; 86.6%) were screened using VIAC. The majority (170/215; 79.1%) were comfortable collecting self-samples for their next cervical cancer screening appointment.

**Table:** DCE participant characteristics

Variable	Participants (N=215), n (%)
**Age (years)**	
Mean age (standard deviation)	37.24 (12.2)
Age groups	
18–29 years	76 (35.3)
30 years and older	139 (64.6)
**Highest education level**	
*Ordinary level	114 (53.0)
*Primary	63 (29.3)
*Tertiary	24 (11.2)
None	9 (4.2)
*Advanced level	5 (2.3)
**Marital status**	
Married	158 (73.5)
Divorced	23 (10.7)
Widowed	20 (9.3)
Single	11 (5.1)
Co-habiting	3 (1.4)
**Employment status**	
Unemployed	117 (54.4)
Employed full-time	57 (26.5)
Employed part-time	41 (19.1)
**Monthly income in USD($)**	
<50	148 (69.2)
50–100	26 (22.15)
100–200	26 (22. 15)
>200	14 (6.5)
**Distance to nearest health facility (km)**	
0–5	113 (52.6)
5–10	51 (23.8)
>10	51 (23.8)
**HPV knowledge (**Have you ever heard of HPV?)	
Yes	185 (86.0)
No	30 (14.0)
**Cervical cancer screening (**Have you been screened for cervical cancer**?)**	
Yes	112 (52.1)
No	103 (47.9)
**Last screening visit for those who were screened** (n=112)	
Less than 5 y ago	94 (83.9)
More than 5 y ago	18 (16.1)
**Method of screening for those who were screened** (n=112)	
VIAC	97 (86.6)
HPV testing	13 (11.6)
PAP smear	2 (1.8)
**Comfortability with HPV self-sampling** (How comfortable are you with the HPV self-sampling for cervical cancer screening?)	
Very comfortable	170 (79.1)
Moderately comfortable	23 (10.7)
Neutral	12 (5.7)
Somewhat comfortable	7 (3.3)
Very uncomfortable	3 (1.4)

⁎ Primary-grade 1-7, ordinary level- form 1-4, advanced level- form 5-6, tertiary-vocational training, college, university.

### Main effects

The fixed effects (Model 1) and mixed effects (Model 3) produced closely similar results in terms of the direction, magnitude, and significance of effects (Supplementary Tables 2 and 3, respectively). Figure shows a forest plot of the ORs of the main effects means for the 215 participants from the mixed effects logit regression model (Model 3). Supplementary Table 4 shows the estimates of the standard deviations (SDs) ORs, *p*-values, and confidence intervals (CIs).

**Figure:**
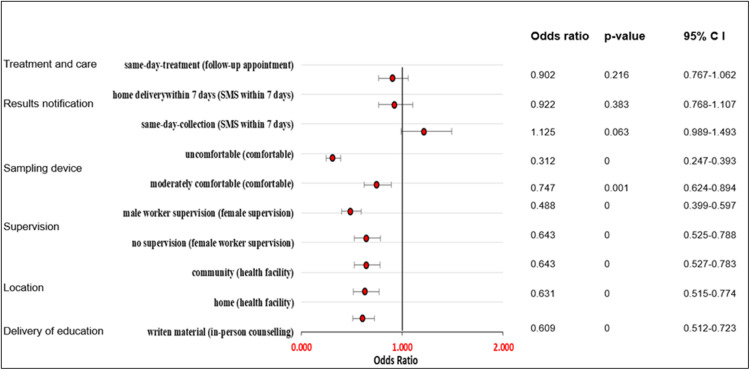
Mixed effects logit model (main effects)

Dzobo. Rural women's preferences for cervical cancer screening via HPV Self-sampling: a discrete choice experiment study in Chidamoyo, Hurungwe District, Zimbabwe. AJOG Glob Rep 2024.

Overall, the participants did not prefer to receive educational information on HPV, cervical cancer and HPV self-sampling through written material compared to in-person counselling or face-to-face education (OR 0.609; 95% CI 0.512–0.723). We found no significant differences in women's preferences for the location of HPV self-sampling between home and community-based self-sampling. Women did not prefer to perform vaginal self-sampling at their homes compared to self-collection at the health facility (OR 0.631; 95% CI 0.515–0.774), nor did they prefer community-based self-sampling (OR 0.643; 95% CI 0.527–0.783) if all the other attributes were held constant. Participants were significantly less likely to perform self-sampling if a male health worker supervised them or if they were unsupervised compared to supervision by a female health worker (OR 0.488; 95% CI 0.399–0.597 and OR=0.643; 95% CI 0.525–0.788). Among the characteristics of an HPV self-sampling delivery approach, the comfort of the sampling device had the most significant impact on women's preferences. Participants were firmly against the use of an uncomfortable sampling device for collecting a vaginal sample compared to a device which felt comfortable if all other attributes remained constant (OR 0.312; 95% CI 0.247–0.393). Although the effect was small, women were less likely to choose a moderately comfortable device compared to a comfortable one for vaginal self-sampling (OR 0.747; 95% CI 0.624–0.894). We found no significant preferences regarding how and when participants received HPV results and care after positive HPV results.

### Interaction effects and stratified analysis

As revealed in Table 3, the SD, ORs, and *p*-values from the mixed-effects logistic regression model indicate preference heterogeneity for some attributes. Supplementary Table 5 presents the interaction analysis results, first showing the differences in preferences between women 18–29 years years and women ≥30 years (Model 4) and then between never-screened women and previously-screened women (Model 5). Preferences were largely similar for women 18–29 years and women ≥30 years. They only differed in preferences regarding methods of results notification and treatment after a positive HPV result, however, the effect was small. Women ≥ 30 years were less likely to prefer home delivery of results (OR=0.823; 95% CI 0.687–0.986) and same-day screen and treatment, respectively (OR=0.816; 95% CI 0.712–0.937). Although preferences between never-screened and previously screened women were primarily consistent, previously screened women were less likely to prioritise same-day screening and treatment (OR=0.843; 95% CI 0.720–0.986).

Stratified models were run to explore each of the four subgroups' preferences in detail. Supplementary Table 6 shows the results (main effects) of the four stratified models for women 18–29 years (Model 6), women ≥ 30 years (Model 7), never-screened women (Model 8), and previously screened women (Model 9). The stratified analysis similarly showed that preferences for women who had never been screened, who had previously been screened, who were 18–29 years, and ≥30 years were largely consistent, as shown by the effects in the same direction. The preference structures for Models 6-9 are also generally consistent with the main effect results from the entire sample, as shown in Figure. This further confirms women's preferences for the different attributes and levels and helps understand the nuances in the attributes where preferences diverge. The results of the stratified analyses revealed that the choice of sampling device was the most significant driver of preferences across all four subgroups (Models 6–9), which confirms the results of the main effects of the full sample. Although preferences were similar across all four subgroups, women aged 30 years and older were less likely to prioritise home delivery of results (OR=0.789; 95% CI 0.627–0.993) and same-day treatment (OR=0.764; 95% CI 0.621–0.941).

### Discussion

This is the first study to investigate women's preferences for HPV self-sampling using the DCE methodology in Zimbabwe. This study revealed significant preferences for health facility self-sampling, female health worker-supervised self-sampling, face-to-face education on cervical cancer and the use of comfortable sampling devices for collecting a vaginal sample. Identifying women's preferences for HPV-based cervical cancer screening via self-sampling will enable the government of Zimbabwe, through the Ministry of Health and Child Care, and relevant stakeholders to make informed decisions when designing cervical cancer screening programmes for nationwide screening.

Overall, this study revealed a general preference for using comfortable and ease-to-use sampling devices compared to sampling devices that were uncomfortable or difficult to use. This finding was the most critical driver of preferences in our study, and it expands on our earlier qualitative findings from a nominal group workshop, where women reported using a metal speculum as a barrier to attending screening.^21^ A systematic review by Nishimura et al. revealed that women preferred a sampling device resembling a basic cotton swab, which they perceived to be comfortable.^28^ Another study by Bishop et al. shows that women preferred an easy-to-use swab that was likely to cause less pain during specimen collection.^29^ Women's acceptability of a sampling device also depends on the availability of easy-to-follow instructions, which are culturally sensitive and in a language most women can understand.^30^ However, we note the need for more evidence from the literature on preferences for sampling devices, especially in LMICs, and this warrants further research on the subject.

Participants in this study preferred health facility-based self-sampling compared to home or community-based self-sampling. Our findings concur with findings from the DCE study that revealed negative preferences for home self-sampling due to reasons ranging from low confidence to performing self-sampling independently and concerns about losing specimens or contaminating them.^23^ However, our findings contradict a systematic review by Nishimura et al., which revealed a stronger preference for home self-sampling among women in high-income countries.^28^ The difference in preferences could be explained by factors such as education level, income, and access to healthcare resources, which can vary across different socio-economic groups and impact women's decisions for self-sampling venues. It is also imperative to note that women's preference for self-sampling at the health facility enables integrated healthcare delivery, which has its own positives, such as cost-cutting by ensuring the health facility is a one-stop shop for sexual and reproductive health services for women. In line with expectations for self-care interventions to increase access to healthcare services, more education is needed to increase the confidence of women to perform self-sampling in the comfort of their homes with little or no help from a health provider. Future studies must investigate the reasons behind women's tradeoffs for the convenience associated with home self-sampling compared to travelling long distances, sometimes over 10km, to access health facility-based self-sampling services.

Additionally, women preferred to receive education and information on cervical cancer, HPV, and self-sampling through face-to-face education or in-person counselling with health workers compared to having written material or infographics. The women's choice for face-to-face interaction with a health worker to receive education on cervical cancer and HPV self-collection may be because at least a third of the women participants did not attain education beyond the primary level and, therefore, would be more comfortable having a health worker explain to them. The dependence of women on health workers and the need to ask further questions could also have been a reason for the preference for face-to-face delivery of educational information, and this corroborates our findings on women's preference for health facility-based self-sampling. Our findings are supported by a qualitative study conducted in South Africa where women were satisfied with verbal explanations by a health provider and did not find added value in receiving information through written material or diagrams.^31^ Based on our findings, it is important to design educational programmes that are culture-sensitive and in local native languages and include community health workers in disseminating the information. Other modes of education delivery, such as radio, can be explored to cater to women who are restricted by distance from accessing the nearest health facilities or who may have missed appointments with health workers teaching about cervical cancer.

Significant value was placed on having female supervision or assistance when performing self-sampling compared to male supervision or having no supervision. This finding illustrates cultural beliefs' influence on women's acceptability and preference for self-sampling screening.^32^ In a scoping review, we revealed that embarrassment was a significant barrier to women participating in cervical cancer screening, and it is reportedly high if the health worker is of the opposite gender.^6^ This is not surprising given the patriarchal nature of the study setting, where women face challenges such as spousal refusal to participate in screening activities.^20^ Given the impact of this characteristic on women's decision-making, there is a need to educate women adequately to improve their confidence and efficacy to perform self-sampling correctly and independently. This is particularly important for understaffed regions to reduce available health workers' workload while maintaining the efficiency of the cervical cancer screening programme.

Based on the main effects results of the total sample, the notification of HPV results and linkage to care and treatment after positive HPV results did not emerge as significant drivers of preferences in our study, seemingly contradicting findings from a DCE study in South Africa, where there was a significant preference for same-day treatment.^11^ The notification of HPV results and linkage to care and treatment after positive HPV results were key characteristics of HPV self-sampling in the qualitative study we conducted earlier.^21^ Therefore we value ensuring timely notification of HPV results to clients and the availability of free and easy-to-access treatment services after an HPV result. The availability of mobile telecommunication services provides an excellent opportunity to inform clients of results and minimise loss to follow-up, which is a significant drawback of cervical cancer screening programmes in LMICs. Another way to mitigate the loss of women to follow-up is same-day treatment by using point-of-care technologies such as benchtop analysers like the Cepheid GeneXpert, which are widely available in Zimbabwe for routine TB and HIV diagnosis.

Our analysis found evidence of preference heterogeneity, which is revealed by differences in preferences. This is an important revelation since it highlights that although the majority of women prioritise specific preference structures, some women will still prefer other delivery approaches for HPV self-sampling. The interaction analysis in our study explored the possibility that the source of the heterogeneity could be the screening experience and age of the participants by looking at the following subgroups: never-screened, previously-screened women, women 18–29 years and women aged 30 years and older. Overall, the interaction analysis results suggest that preference structures were largely homogeneous among never-screened, previously screened, young and older women. However, young women (18–29 years) and never screened women favoured same-day screening and treatment compared to older women (≥30 years) and women with screening experience. This could be explained by the fact that most women who are 18–29 years old are ineligible for screening under the current screening guidelines in Zimbabwe and, therefore, consider HPV testing and same-day screening to be an acceptable screening option when they are eligible for cervical cancer screening. This is not the case for older women who are used to VIAC-based cervical cancer screening. Given the proven benefits of HPV testing via self-sampling and the added advantage of same-day treatment, it is essential to educate women of all ages on HPV testing via self-sampling to ensure increased demand for the intervention, which is critical to increasing screening coverage. The stratified models for screening experience and age were largely consistent, showing that the choice of sampling device was the most significant factor driving women's preferences for HPV self-sampling in rural Zimbabwe. It is possible that preference heterogeneity was due to other factors not explored in this study. Future studies must investigate this further to explore other sources of heterogeneity, such as level of education and income status.

Moreover, when viewed through the lens of the Socio-ecological model, our study findings offer significant insights into women's decision-making process, highlighting the interplay of factors in determining acceptable delivery approaches for HPV self-sampling. At the Individual level, women prioritised health-facility-based self-sampling compared to self-sampling at home, which may reflect the lack of expertise or confidence to perform the procedure independently. It may also reveal women's trust in the capabilities of health workers, which aligns with the interpersonal construct of the Socio-ecological model. Women's preference for a comfortable sampling device aligns with the individual construct of the Socio-ecological model, which highlights women's willingness to perform self-sampling correctly if the device is comfortable. The study findings revealed a preference for female supervision compared to male supervision, highlighting the Socio-ecological model's intrapersonal and community domains. The feeling of embarrassment when supervised by a male health worker and the unwillingness of most male spouses to have their wives exposed to another male may have prompted women to prefer female supervision.

From the demand side, women made significant tradeoffs against attributes such as results notification and linkage to care, which align more with the health systems domain of the Socio-ecological model, representing the supply side of a cervical cancer screening intervention. These findings demonstrate that, although user preferences are essential in designing interventions, there is the supply side of the intervention, which needs the input of the programme managers and policymakers. In this case, policymakers should create programmes that ensure timely results notification, particularly same-day screen and treat programmes, which have been found to reduce the number of women lost to follow-up and improve programme efficiencies at the population level.^33^

### Strengths and limitations

The findings of this study demonstrate the utility of a DCE methodology to elicit information about consumers' ability to make tradeoffs about different characteristics of healthcare services to align with their preferences. This study revealed the importance of understanding preferences for HPV self-sampling among rural women that are traditionally disadvantaged due to socio-cultural factors which limit their access to and use of sexual reproductive health services. However, limiting the study to rural women in Chidamoyo, Hurungwe, means that the study findings, though useful, may not be generalisable to women in other rural contexts and urban women. Further research is needed to understand the preferences of women in non-rural contexts. We followed one of the recommended rules of thumb to calculate an adequate sample size to draw meaningful conclusions. However, the participants were not randomly selected and may not represent the study population. To mitigate the non-randomness of the sample, we randomly assigned participants to the four versions of the DCE design. This study used a design without an opt-out option to maximise the information about participant tradeoffs. Therefore, our results are only necessary for understanding overall preferences for HPV self-sampling delivery strategies but cannot be used as a predictor for demand since there was no reliable anchor for willingness to perform self-sampling for cervical cancer screening. We did not include cost as an attribute for our DCE, considering how it impacts the real-world tradeoffs individuals face when making decisions. This is so because the majority of sexual and reproductive health programmes in rural Zimbabwe, including HIV services, are offered for free at government institutions.

## Conclusion

This research highlights the importance of understanding the drivers of choice when designing interventions that will facilitate the uptake of cervical cancer screening. This is particularly important in Zimbabwe, where the Ministry of Health and Child Care is introducing HPV testing for primary cervical cancer screening. Our study augments the WHO's call for self-care interventions to promote health equality among women. Key findings from this study emphasise the importance of educating women on cervical cancer and screening methods and using acceptable sampling devices that are comfortable and easy to use to promote the uptake of self-sampling screening. This study emphasises how patients depend on healthcare workers for most of their healthcare needs. Educating and raising awareness among patients about using self-care services, especially in resource-constrained settings like Zimbabwe, is crucial to promote task shifting. Further research is needed to understand how the preference for cervical cancer screening through self-sampling translates into real-world uptake.

## Appendix Supplementary materials

Image, application 1

## Footnotes

Supplementary material associated with this article can be found in the online version at doi:10.1016/j.xagr.2024.100414.
