# Supplementary material for: Rural women's preferences for cervical cancer screening via HPV self-sampling: a discrete choice experiment study in chidamoyo, Hurungwe District, Zimbabwe
Source: AJOG Glob Rep. 2024 Oct 17;4(4):100414. doi: 10.1016/j.xagr.2024.100414 (PMC11570521; doi:10.1016/j.xagr.2024.100414)
Supplement: Supplementary file 1 [file mmc1.pdf]

Supplementary for “Rural Women's Preferences for Cervical Cancer Screening via HPV Self-Sampling: A Discrete Choice Experiment in Chidamoyo, Hurungwe District, Zimbabwe”

Supplementary Tables

Supplementary Table 1: Discrete choice experiment-attributes and levels and description

Attributes	Levels	Description
Location for performing self-sampling	1. Homestead<br>2. Health facility<br>3. Community	1. This means health workers will deliver the sampling kit to your home, or you can collect the sampling kit at the nearest health facility and perform self-sampling in the comfort of your home. After collection, you can either take the sample to the health facility, or it will be collected by community health workers or designated transport systems<br>2. This means that you will visit the health facility, and a health provider will offer you a sampling kit to collect your sample in a private room at the health facility<br>3. This means you will be invited to a community gathering, usually consisting of mobile health units close to your home where health workers will give you a sampling kit to collect a sample in a private space.
Education and Information	1. In-Person Counseling<br>2. Educational Materials	1. Women receive face-to-face counselling from healthcare providers, including community health workers, about HPV, self-sampling, cervical cancer, screening process and treatment and management options after a positive HPV result<br>2. Women receive written or audiovisual materials that provide detailed instructions and information about HPV, self-sampling, cervical cancer, screening process and treatment and management options after a positive HPV result
Supervised self-sampling	1. No supervision<br>2. Supervision by a female health worker<br>3. Supervision by a male health worker	1. Means no one is present during the self-sampling process. The participant has complete privacy and performs the sampling without any assistance or supervision<br>2. A female health worker is present during the self-sampling process. This is for women who need support and reassurance to feel comfortable and confident, but only with health workers of the same gender.<br>3. This option considers women who need support and assurance during the self-sampling process but do not mind the gender of the health worker.
The comfort of the sampling device	1. Easy and comfortable<br>2. Moderately easy and comfortable<br>3. Difficult and uncomfortable to	1. This level could represent a sampling device that is designed to be user-friendly. It may involve minimal or no discomfort at all<br>2. This level could represent a relatively easy-to-use sampling device that may involve some minor discomfort or inconvenience.<br>3. This describes a sampling device that is challenging to use, potentially causing discomfort or inconvenience during the sampling process. It may require additional effort or demonstration by health workers

When and how you receive HPV results	1. Collection at the health facility on the same day of self-sampling-same day collection at the health facility<br>2. Text-based messaging (SMS)-within 7 days of performing self-sampling<br>3. Home delivery within 7 days of self-sampling	1. You will get your results within 4 hours at the health facility on the same day of performing self-sampling.<br>2. You will get your results within 7 days of performing self-sampling; results will be sent to your phone through an SMS<br>3. within 7 days of conducting self-sampling, results will be delivered to your homestead in a sealed envelope for confidentiality
Care and treatment after positive HPV result	1. Same-day screening and treatment<br>2. Follow-up appointment at the health facility	1. When tested for HPV and you are found HPV positive, you will be treated or get further management on the same day<br>2. If you are HPV positive, you will be invited to come back to the health facility on a different day for treatment or further management.

Acronyms: OR-Odds ratio, CI-confidence interval, HPV-Human papillomavirus SMS-short message service

Supplementary Table 2: Fixed effects conditional logit (Model 1)

Attribute	Attribute level (Reference category)	OR	p-value	95% CI
Education delivery method	Written material (in-person counselling)	0.671	0	0.601-0.748
Location of self-sampling	Home (health facility)	0.689	0	0.593-0.800
	Community (health facility)	0.707	0	0.609-0.820
Supervision of HPV self-sampling	No supervision (female supervision)	0.704	0	0.609-0.814
	Male supervision (female supervision)	0.569	0	0.491-0.660
Comfort of sampling device	Moderately comfortable (comfortable)	0.800	0.002	0.693-0.922
	Uncomfortable (comfortable)	0.419	0	0.360-0.487
Results notification	Same-day-collection (SMS within 7 days)	1.160	0.047	1.001-1.343
	Home delivery within 7 days (SMS within 7 days)	0.936	0.367	0.811-1.080
Treatment after positive HPV result	Same-day-treatment (follow-up appointment)	0.930	0.186	0.834-1.036

Acronyms: OR-Odds ratio, CI-confidence interval, HPV-Human papillomavirus SMS-short message service

Supplementary Table 3: Mixed effects logit (Model 2) main effects

Attribute	Attribute level (Reference category)	OR	p-value	95% CI
Education delivery method	Written material (in-person counselling)	0.609	0	0.512-0.723
Location of self-sampling	Home (health facility)	0.631	0	0.515-0.774
	Community (health facility)	0.643	0	0.527-0.783
Supervision of HPV self-sampling	No supervision (female supervision)	0.643	0	0.525-0.788
	Male supervision (female supervision)	0.488	0	0.399-0.597
Comfort of sampling device	Moderately comfortable (comfortable)	0.747	0.001	0.624-0.894
	Uncomfortable (comfortable)	0.312	0	0.247-0.393
Results notification	Same-day-collection (SMS within 7 days)	1.215	0.063	0.989-1.493
	Home delivery within 7 days (SMS within 7 days)	0.922	0.383	0.768-1.107
Treatment after positive HPV result	Same-day-treatment (follow-up appointment)	0.902	0.216	0.767-1.062

Acronyms: OR-Odds ratio, CI-confidence interval, HPV-Human papillomavirus SMS-short message service

Supplementary Table 4: Mixed effects logit (Model 3) standard deviations

Attribute	Attribute level (Reference category)	OR	p-value	95% CI
Education delivery method	Written material (in-person counselling)	1.990	0	1.551-2.553
Location of self-sampling	Home (health facility)	1.567	0.049	1.002-2.450
	Community (health facility)	1.706	0.004	1.189-2.448
Supervision of HPV self-sampling	No supervision (female supervision)	1.896	0	1.384-2.597
	Male supervision (female supervision)	1.598	0.017	1.088-2.348
Comfort of sampling device	Moderately comfortable (comfortable)	1.001	0.994	0.753-1.331
	Uncomfortable (comfortable)	0.988	0.96	0.607-1.606
Results notification	Same-day-collection (SMS within 7 days)	1.984	0	1.427-2.758
	Home delivery within 7 days (SMS within 7 days)	0.970	0.909	0.577-1.630
Treatment after positive HPV result	Same-day-treatment (follow-up appointment)	1.643	0.001	1.220-2.213

Acronyms: OR-Odds ratio, CI-confidence interval, HPV-Human papillomavirus SMS-short message service

Supplementary Table 5: Analysis of interaction between groups

		Model 4			Model 5		
		**Women 18-29 years**			**Never screened women**		
**Attribute**	**Level (Reference category)**	**OR**	**p-value**	**95% CI**	**OR**	**p-value**	**95% CI**
Education delivery method	Written material (in-person counselling)	0.707	0	0.589-0.849	0.672	0	0.576-0.785
Location of self-sampling	Home (health facility)	0.815	0.105	0.636-1.044	0.786	0.025	0.637-0.970
	Community (health facility)	0.826	0.137	0.643-1.062	0.830	0.089	0.670-1.029
Supervision of HPV self-sampling	No supervision (female supervision)	0.684	0.002	0.535-0.874	0.699	0.001	0.567-0.862
	Male supervision (female supervision)	0.604	0	0.471-0.773	0.637	0	0.516-0.786
Comfort of sampling device	Moderately comfortable (comfortable)	0.811	0.09	0.637-1.033	0.888	0.26	0.723-1.092
	Uncomfortable (comfortable)	0.419	0	0.326-0.538	0.463	0	0.374-0.574
Results notification	Same-day-collection (SMS within 7 days)	1.353	0.016	1.057-1.731	1.321	0.01	1.070-1.632
	Home delivery within 7 days (SMS within 7 days)	1.181	0.178	0.927-1.505	1.039	0.718	0.845-1.277
Treatment after positive HPV result	Same-day-treatment (follow-up appointment)	1.175	0.082	0.980-1.409	1.047	0.562	0.897-1.220
		**Women ≥ 30 years**			**Previously screened women**		
Education delivery method	Written material (in-person counselling)	0.648	0	0.565-0.743	0.660	0	0.565-0.772
Location of self-sampling	Home (health facility)	0.623	0	0.516-0.752	0.601	0	0.484-0.745
	Community (health facility)	0.645	0	0.535-0.777	0.604	0	0.489-0.745
Supervision of HPV self-sampling	No supervision (female supervision)	0.711	0	0.593-0.852	0.691	0	0.563-0.847
	Male supervision (female supervision)	0.544	0	0.451-0.656	0.497	0	0.402-0.615
Comfort of sampling device	Moderately comfortable (comfortable)	0.789	0.01	0.659-0.944	0.714	0.001	0.582-0.875
	Uncomfortable (comfortable)	0.413	0	0.341-0.501	0.372	0	0.298-0.463
Results notification	Same-day-collection (SMS within 7 days)	1.075	0.444	0.894-1.292	1.022	0.836	0.831-1.258
	Home delivery within 7 days (SMS within 7 days)	0.823	0.035	0.687-0.986	0.854	0.126	0.697-1.045
Treatment after positive HPV result	Same-day-treatment (follow-up appointment)	0.816	0.004	0.712-0.937	0.843	0.032	0.720-0.986

Acronyms: OR-Odds ratio, CI-confidence interval, HPV-Human papillomavirus SMS-short message service

Supplementary Table 6: Models 6-9 main effects stratified by screening experience and age Odds ratios and p-values

		Never-screened (Model 6)		Previously screened (Model 7)		18-29 years (Model 8)		≥ 30years (Model 9)	
**Attribute (reference)**	**Level**	**OR**	**p-value**	**OR**	**p-value**	**OR**	**p-value**	**OR**	**p-value**
Education delivery method (in-person counselling)	Written material	0.612	0	0.572	0	0.656	0.006	0.576	0
Location of self-sampling (health facility)	Home	0.704	0.023	0.547	0	0.725	0.061	0.578	0
	Community	0.785	0.081	0.510	0	0.771	0.138	0.575	0
Supervision of HPV self-sampling (female supervision)	No supervision	0.616	0.001	0.624	0.002	0.616	0.006	0.661	0.001
	Male supervision	0.545	0	0.405	0	0.512	0	0.466	0
Comfort of sampling device (comfortable)	Moderately comfortable	0.847	0.202	0.640	0.001	0.747	0.061	0.734	0.008
	Uncomfortable	0.360	0	0.246	0	0.309	0	0.306	0
Results notification (SMS within 7 days)	Same-day-collection	1.467	0.014	1.013	0.933	1.509	0.022	1.099	0.472
	Home delivery	1.081	0.565	0.790	0.093	1.248	0.164	0.789	0.044
Treatment after positive HPV result (follow-up appointment)	Same-day-treatment	1.031	0.784	0.777	0.059	1.176	0.237	0.764	0.011

Acronyms: OR-Odds ratio, CI-confidence interval, HPV-Human papillomavirus SMS-short message service

Supplementary Figures

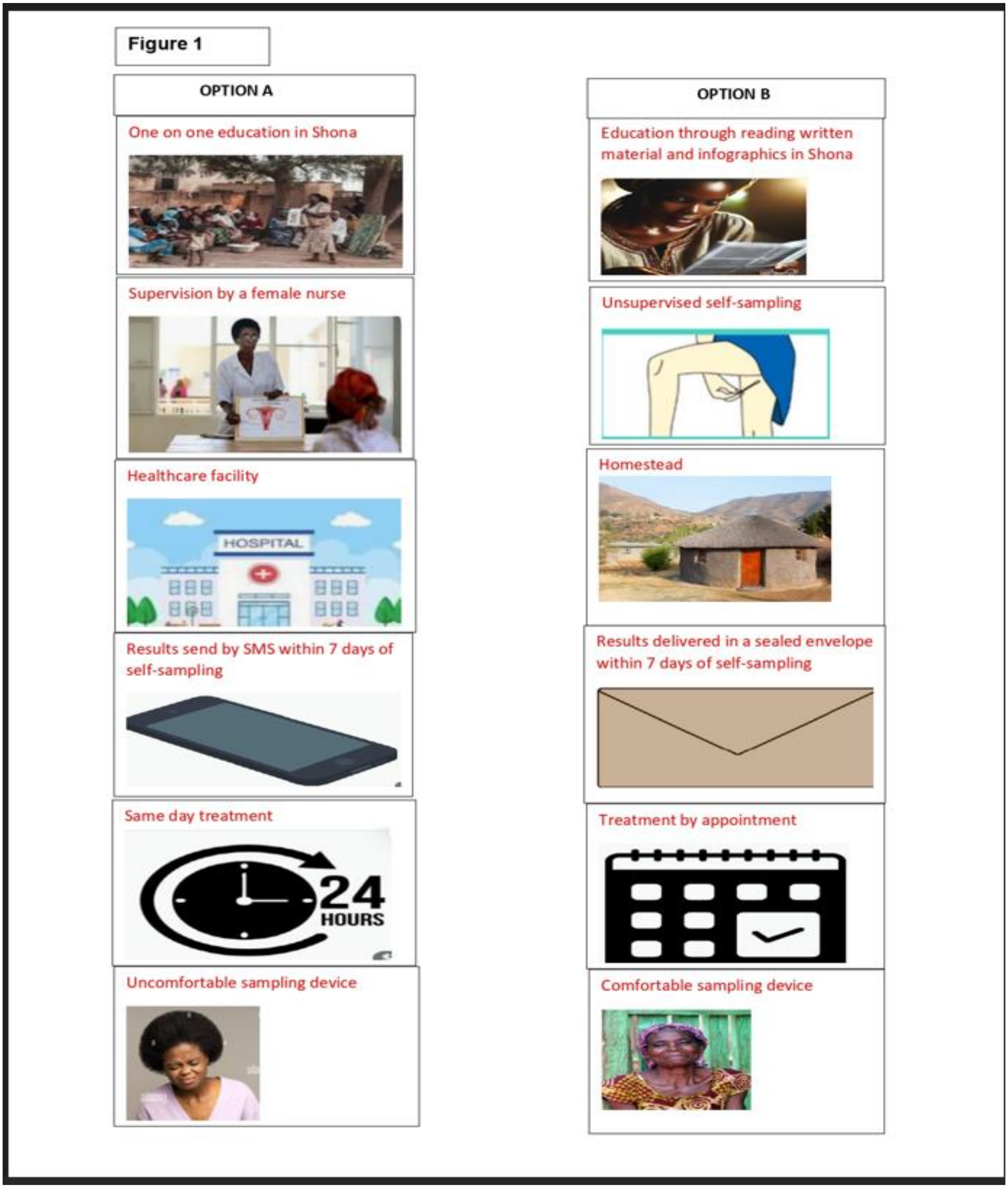

Supplementary Figure 1: An example of a choice set for an HPV self-sampling delivery approach
